# Clinical Evaluation of HHLA2 in Hormone Receptor-Positive Breast Cancer: Expression Patterns and Prognostic Implications

**DOI:** 10.3390/biomedicines14092066

**Published:** 2026-09-15

**Authors:** Mi Jung Kwon, Joo Mi Yi, Tae Hyun Kim, Ha Young Park

**Affiliations:** 1 Department of Pathology, Hallym University Sacred Heart Hospital, Hallym University College of Medicine, Anyang 14068, Republic of Korea; 2 Department of Microbiology and Immunology, Inje University College of Medicine, Busan 47392, Republic of Korea; 3 Cardiovascular and Metabolic Diseases Medical Research Center, Inje University College of Medicine, Busan 47392, Republic of Korea; 4 Department of Surgery, Busan Paik Hospital, Inje University College of Medicine, Busan 47392, Republic of Korea; 5 Department of Pathology, Busan Paik Hospital, Inje University College of Medicine, Busan 47392, Republic of Korea

**Keywords:** HHLA2, B7 family, immune checkpoint, hormone receptor-positive breast cancer, prognosis, immunohistochemistry, TCGA

## Abstract

**Background:** HHLA2 (human endogenous retrovirus-H long terminal repeat-associating protein 2) is a B7 family immune checkpoint molecule that delivers costimulatory or coinhibitory signals depending on the receptor engaged. Although it has been implicated in various cancers, its clinical significance in hormone receptor (HR)-positive breast cancer remains unclear. **Methods:** Ninety-three patients with HR-positive breast cancer who underwent surgical resection were analyzed using tissue microarrays. HHLA2 expression was assessed by immunohistochemistry using a combined intensity and proportion score (0–8), analyzed as a continuous variable by Cox regression, with dichotomized comparisons (score >5 vs. ≤5) as sensitivity analyses. Associations with clinicopathologic variables were evaluated using Fisher’s exact test and Spearman correlation. A complementary analysis was performed using RNA-sequencing data from the TCGA-BRCA cohort. **Results:** HHLA2 immunoreactivity was detected in 86 tumors (92.5%). No significant associations were identified between HHLA2 expression and age, tumor size, nodal status, grade, or HER2 status. The HHLA2 score was not associated with overall, disease-specific, or relapse-free survival (hazard ratio per 1-point increase 1.17, 1.86, and 1.19, respectively; all *p* > 0.1), and no cutoff reached significance after correction for multiple comparisons. In TCGA-BRCA, HHLA2 transcript levels were low overall and higher in triple-negative than in HR-positive tumors (*p* < 0.001), but were not associated with survival in either group. **Conclusions:** HHLA2 immunoreactivity was detected in most HR-positive breast cancers, but no association with clinicopathologic features or survival was detected in this small exploratory cohort. Larger studies are needed to exclude modest prognostic effects.

## 1. Introduction

Human endogenous retrovirus-H long terminal repeat-associating protein 2 (HHLA2), also known as B7-H7, is an emerging immune checkpoint molecule belonging to the B7 family [1]. Unlike conventional B7 family members that primarily deliver either stimulatory or inhibitory signals, HHLA2 exhibits unique bidirectional immunomodulatory properties, acting on T and natural killer (NK) cells through two distinct receptors. It delivers costimulatory signals through TMIGD2 (CD28H) on naïve T and NK cells, whereas engagement of KIR3DL3 on activated T and NK cells delivers coinhibitory signals [1,2]. HHLA2 expression has been documented across multiple solid malignancies, including lung cancer [3,4], pancreatic ductal adenocarcinoma [5], clear cell renal cell carcinoma [6,7], gastric cancer [8], and triple-negative breast cancer (TNBC) [9]. The prognostic significance of HHLA2 varies across tumor types, with some studies reporting associations with adverse outcomes while others demonstrate neutral or favorable effects. These context-dependent findings, combined with the dual immunomodulatory functions of HHLA2, suggest complex roles in tumor immunity and potential value as both a prognostic biomarker and therapeutic target [1].

While immune checkpoints such as PD-1, PD-L1, and CTLA-4 have been extensively characterized in breast cancer, HHLA2 remains comparatively understudied, with conflicting evidence regarding its biological and clinical significance [10]. The most comprehensive breast cancer-specific study to date, by Janakiram et al., examined TNBC and found HHLA2 upregulation in 56% of early-stage cases, with high expression associated with advanced stage and lymph node metastasis [9]. However, studies in other cancer types have reported opposite prognostic associations, with HHLA2 expression linked to improved outcomes [11,12]. These contradictory findings underscore the context-dependent nature of HHLA2 in tumor immunity. In hormone receptor (HR)-positive breast cancer, which accounts for the majority of cases, the expression pattern and clinical significance of HHLA2 remain essentially unexplored.

The net effect of HHLA2 likely depends on the relative availability of its two receptors within a given tumor microenvironment [1]. This is particularly relevant in breast cancer, where molecular subtypes differ markedly in immune composition. HR-positive tumors are characterized by sparse immune infiltration and an immunologically “cold” microenvironment [13], in contrast to the more immune-enriched landscape of TNBC. The functional role and clinical significance of HHLA2 may therefore differ between these subtypes. However, whether HHLA2 is expressed by tumor cells in HR-positive breast cancer, and whether its expression carries prognostic information in this immunologically quiescent setting, has not been examined.

We therefore examined HHLA2 protein expression in HR-positive breast cancer using tissue microarray-based immunohistochemistry, and assessed its associations with clinicopathologic parameters and survival. To place these findings in a broader molecular context, we analyzed HHLA2 transcript levels in The Cancer Genome Atlas breast cancer dataset (TCGA-BRCA), comparing expression across molecular subtypes and assessing prognostic associations in the HR-positive and triple-negative groups.

## 2. Materials and Methods

### 2.1. Patient Cohort and Tissue Samples

We retrospectively reviewed HR-positive breast cancer cases diagnosed and surgically treated at Busan Paik Hospital between 2004 and 2010. All patients were female. Tumors were classified as HR-positive when ER and/or PR was positive by immunohistochemistry, irrespective of HER2 status. Intrinsic subtyping (PAM50) was not available. Tissue microarrays (TMAs) constructed for a previous study were used; each case was represented by a single 2 mm core taken from a representative tumor area of a formalin-fixed, paraffin-embedded (FFPE) block, with 22–24 cores per block. Of 126 patients, 93 were included; the remaining 33 were excluded because the tumor core was lost or damaged during sectioning of these archival TMAs, leaving insufficient tissue for interpretation. Approval for this study was obtained from the Institutional Review Board of Busan Paik Hospital (IRB No. 17-0195).

### 2.2. Immunohistochemistry and Scoring for HHLA2

HHLA2 immunostaining was performed using a rabbit polyclonal anti-HHLA2 antibody (catalog no. PA5-24146; Thermo Fisher Scientific, Rockford,IL, USA; 1:250 dilution) on the Ventana Benchmark ULTRA platform (Ventana Medical Systems, Tucson, AZ, USA) following the manufacturer’s instructions. Normal colonic mucosa stained with the same antibody, dilution and platform during antibody optimization served as an external positive control (Figure S1). HHLA2 staining in tumor cells was cytoplasmic and granular with membranous accentuation; membranous and/or cytoplasmic staining was considered positive, and the two components were not scored separately. Tumor stroma and endothelium were consistently negative and served as internal negative controls.

HHLA2 expression was semi-quantitatively assessed by combining staining intensity (0, none; 1, weak; 2, moderate; 3, strong) and the percentage of positive tumor cells (0, none; 1, <1%; 2, 1–10%; 3, 11–33%; 4, 34–66%; 5, 67–100%), resulting in a total score of 0–8. Slides were scored independently by two pathologists blinded to clinical outcomes; discordant cases were resolved by joint review at a multi-headed microscope. Individual pre-consensus scores were not retained, and interobserver agreement was therefore not calculated.

**Figure 1:**
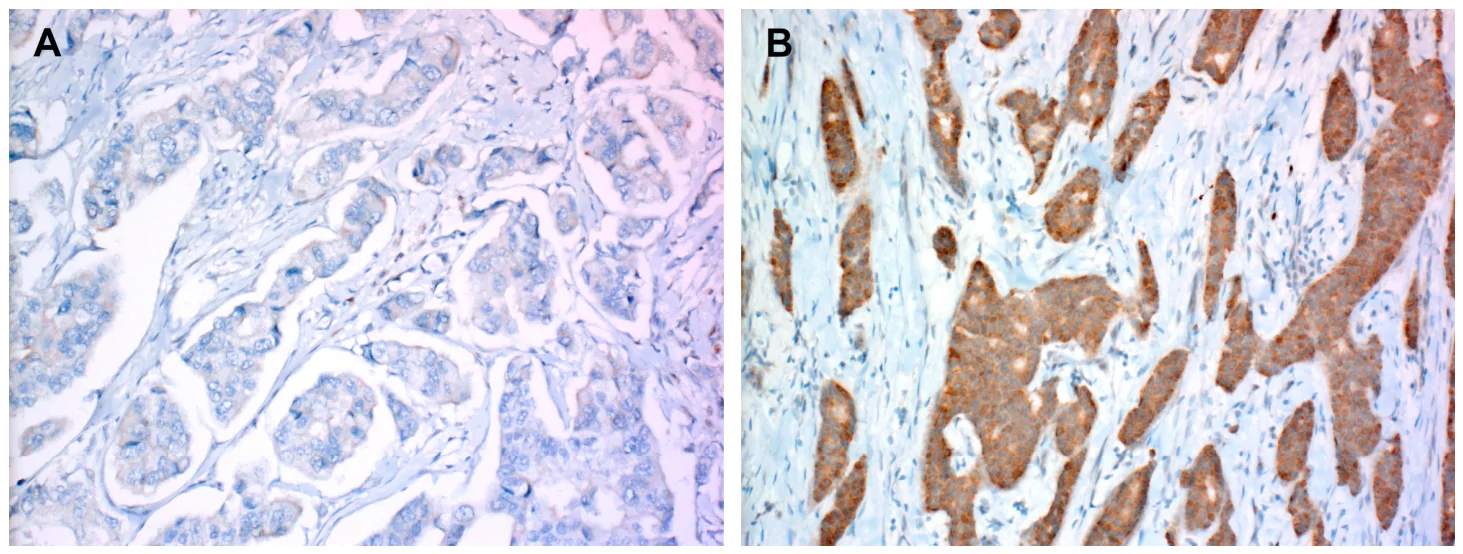
Representative immunohistochemical staining of HHLA2 in hormone receptor-positive breast cancer tissue microarrays (TMAs). (**A**) Low HHLA2 expression showing weak or absent staining in tumor cells. (**B**) High HHLA2 expression showing strong and diffuse membranous or cytoplasmic staining in tumor cells. Total expression scores (0–8) in tumor cells, combining intensity and proportion, were classified as low (≤5, *n* = 33) or high (>5, *n* = 60). Original magnification, ×200.

The total score was analyzed primarily as a continuous variable. For descriptive comparisons, cases were additionally dichotomized at a total score > 5 (high) versus ≤5 (low) (Figure 1); this cutoff was one of five candidate values (>3 to >7) examined, none of which reached significance for any survival endpoint after correction for multiple comparisons (Table S1).

### 2.3. Clinicopathologic Variables

Clinicopathologic information, including age, tumor size, nodal status, histologic grade, ER, PR, HER2 status, and survival data, was obtained from electronic medical records. All cases were staged according to the AJCC Cancer Staging Manual, 6th edition, which was in use during the majority of the study period. ER and PR positivity were scored according to the Allred scoring system, with a total score ≥ 3 considered positive. HER2 status was assessed by immunohistochemistry and interpreted according to the criteria in use at the time of diagnosis; in situ hybridization was not performed during the study period. Tumors with a score of 3+ were classified as HER2-positive and those with a score of 0 or 1+ as HER2-negative. No tumor in the cohort showed a 2+ result.

Overall survival (OS) was defined as the interval from the date of surgery to death from any cause. Disease-specific survival (DSS) was defined as the interval from surgery to death from breast cancer, with deaths from other causes censored. Relapse-free survival (RFS) was defined as the interval from surgery to the first documented recurrence; deaths without recurrence were not counted as events. Vital status and cause of death were obtained from the national death registry (Statistics Korea). As recurrence data were available only up to the last clinical visit, RFS was censored at that date. For OS and DSS, patients without an event were censored at the date of last follow-up. All intervals were expressed in months.

### 2.4. TCGA Data Acquisition and Analysis

RNA-sequencing expression profiles and clinical annotations from the TCGA-BRCA cohort were obtained from the Genomic Data Commons (GDC) portal (https://portal.gdc.cancer.gov/; accessed on 18 April 2024). Gene expression values for HHLA2 were obtained as unstranded transcripts per million (TPM) values from the STAR-Counts workflow (GENCODE v36 gene model). For comparison of HHLA2 expression across tumor types, RNA-seq profiles of 23 other epithelial solid tumors were also retrieved (TCGA project codes: ACC, BLCA, CESC, CHOL, COAD, ESCA, HNSC, KICH, KIRC, KIRP, LIHC, LUAD, LUSC, MESO, OV, PAAD, PRAD, READ, STAD, THCA, THYM, UCEC, and UCS). Of the 1230 RNA-seq files retrieved for the TCGA-BRCA cohort, 16 duplicate primary tumor files were excluded so that each case contributed one sample, retaining the file from the earliest vial; where vials were identical, the file with the lowest GDC file UUID was retained. Five further samples from four cases flagged for redaction in the TCGA Pan-Cancer Clinical Data Resource [14] were also excluded. The final set comprised 1209 samples (1090 primary tumors, 112 normal tissues, and 7 metastatic samples). For the pan-cancer comparison all primary tumor files were used without filtering (*n* = 8434 across 24 tumor types).

Molecular subtypes were assigned based on hormone receptor (HR) and HER2 status recorded in the TCGA clinical data. HR status was taken from the recorded ER and PR results. HER2 status was assigned hierarchically: HER2/CEP17 ratio (≥2.0, positive), then reported FISH status, then IHC score (3+, positive; 0 or 1+, negative); 2+ results were not used. Where none was available, tumors were called negative only if the curated TCGA field recorded a negative result, and cases recorded as positive without supporting evidence were treated as indeterminate. Tumors were classified as HR+/HER2−(ER- and/or PR-positive, HER2-negative), HR+/HER2+ (ER- and/or PR-positive, HER2-positive), HER2+ (ER- and PR-negative, HER2-positive), or triple-negative (ER-, PR-, and HER2-negative). Of the 1090 primary tumors, 914 with a definitive subtype were included; 176 were excluded because hormone receptor status was missing (*n* = 52) or HER2 status could not be established (*n* = 124). For survival analyses, HHLA2 expression was analyzed primarily as a continuous variable (log2[TPM + 1], standardized within each cohort and expressed per SD). Because transcript levels were near the detection floor (median 0.027 TPM; 29.9% of primary tumors with zero mapped reads), detectable (TPM > 0) versus undetectable expression was examined as the principal dichotomized sensitivity analysis, with median and upper-quartile splits as further alternatives (Table S2). To assess whether detectability depended on sequencing depth (total gene-mapped reads per sample), depth was compared across subtypes, and detectability was modeled by logistic regression with TN status and log10 depth as covariates. Overall survival (OS) and progression-free interval (PFI) were obtained from the same resource [14]. PFI was defined as the interval from initial diagnosis to the first new tumor event—progression, locoregional recurrence, distant metastasis, or new primary tumor—or death with tumor, with all other patients censored at the date of last follow-up.

### 2.5. Statistical Analysis

Analyses were performed using Python 3.13.14 with lifelines 0.30.3, scipy 1.18.0, scikit-posthocs 0.14.0, and statsmodels 0.14.6; figures were generated using matplotlib 3.11.0 and seaborn 0.13.2.

In the study cohort, continuous variables were compared using the Mann–Whitney U test and categorical variables using Fisher’s exact test; ordered categorical variables (stage, histologic grade) were compared using the linear-by-linear association test. Associations between the HHLA2 score and clinicopathologic parameters were assessed by Spearman’s rank correlation. Overall survival, disease-specific survival, and relapse-free survival were estimated by the Kaplan–Meier method. The HHLA2 total score was analyzed primarily as a continuous variable (per 1-point increase) by univariable Cox proportional hazards regression. Dichotomized comparisons at each candidate cutoff (>3 to >7) were performed by the log-rank test as sensitivity analyses, with Bonferroni correction for five comparisons per endpoint. Multivariable analysis was not performed because of the small number of events.

In the TCGA cohort, HHLA2 expression was compared across groups by the Kruskal–Wallis test, followed by Dunn’s post hoc test with Benjamini–Hochberg correction when the overall test was significant. Sequencing depth was compared across subtypes by the Kruskal–Wallis test, and detectability was modeled by logistic regression with subtype and depth as covariates. Overall survival and progression-free interval were analyzed by univariable Cox proportional hazards regression for the continuous expression variable and by the Kaplan–Meier method with the log-rank test for dichotomized comparisons.

All tests were two-sided, and *p* < 0.05 was considered statistically significant. Given the number of comparisons performed and the limited number of events, survival analyses were considered exploratory.

## 3. Results

### 3.1. HHLA2 Expression and Clinicopathologic Characteristics in Breast Cancer

The study cohort consisted of 93 patients with HR-positive breast cancer who underwent surgical resection. HHLA2 immunoreactivity was detected in 86 of 93 tumors (92.5%). Using a total score above 5, 60 patients (64.5%) were classified as HHLA2-high and 33 (35.5%) as HHLA2-low (Figure 1).

Baseline characteristics were compared between the two groups (Table 1). Age was comparable, with a median of 54 years (IQR, 46–61) in the HHLA2-high group and 56 years (IQR, 49–61) in the HHLA2-low group (*p* = 0.329). No significant differences were observed in tumor size (>2 cm in 50.0% vs. 36.4%; *p* = 0.277), lymph node metastasis (present in 51.7% vs. 42.4%; *p* = 0.516), or HER2 status (positive in 13.3% vs. 9.1%; *p* = 0.741). Stage and histologic grade, compared across their ordered categories, also did not differ significantly (*p* = 0.267 and *p* = 0.148, respectively). Although none of these comparisons reached statistical significance, the directions were not uniform: HHLA2-high tumors were numerically larger, more often node-positive, and more often of higher stage, whereas high-grade tumors were less frequent in this group.

To assess these associations using the HHLA2 score as a continuous measure rather than a dichotomized variable, Spearman rank correlation was performed (Table 2). No correlation reached statistical significance. Correlations with tumor size (ρ = 0.079, *p* = 0.451), lymph node metastasis (ρ = 0.059, *p* = 0.572), stage (ρ = 0.054, *p* = 0.610), and HER2 status (ρ = 0.027, *p* = 0.800) were negligible, while histologic grade showed a weak inverse correlation (ρ = −0.143, *p* = 0.172), consistent with the grade distribution in Table 1. Taken together, HHLA2 expression in HR-positive breast cancer was not significantly associated with any of the standard clinicopathologic prognostic parameters examined.

### 3.2. Survival Analysis of HHLA2 Expression in Breast Cancer Patients

**Figure 2:**
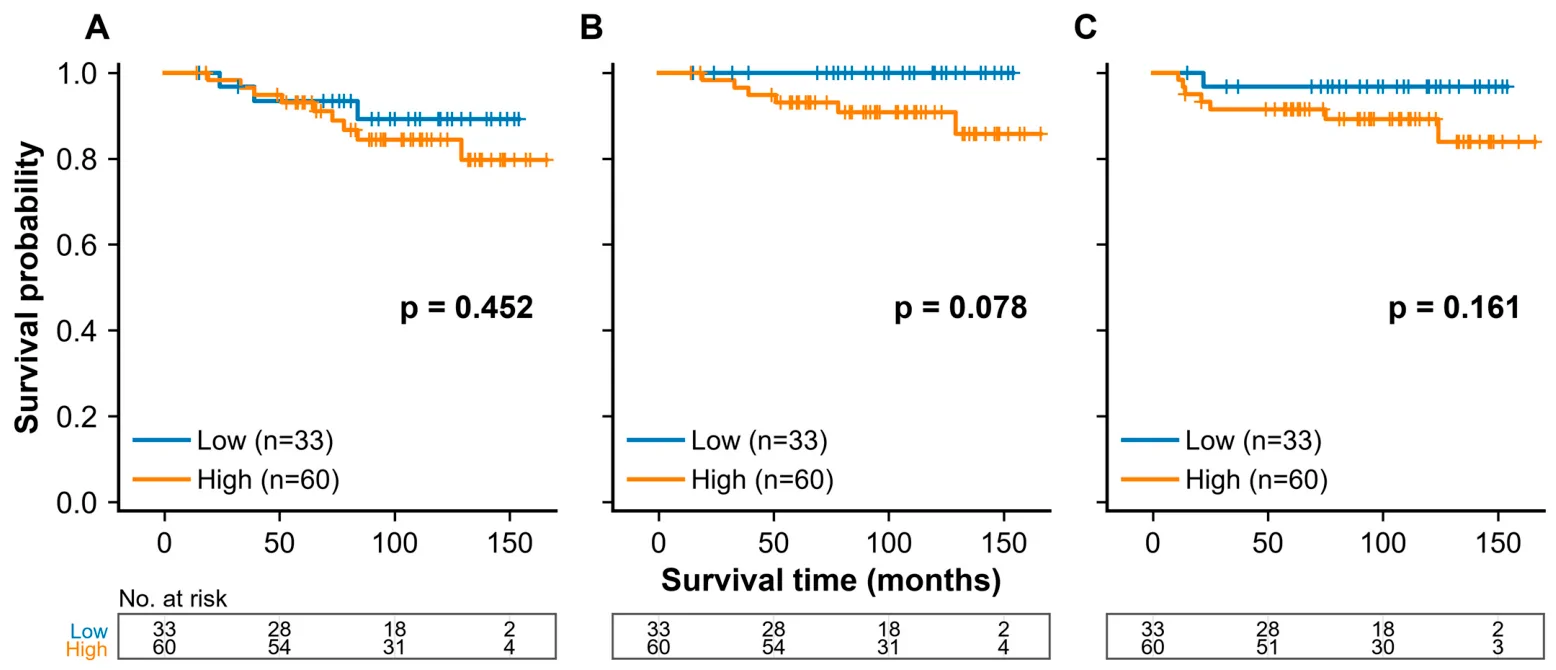
Kaplan–Meier survival analysis according to HHLA2 expression in hormone receptor-positive breast cancer. (**A**) Overall survival (OS), (**B**) disease-specific survival (DSS), and (**C**) relapse-free survival (RFS) in the HHLA2-high (total immunohistochemical score > 5, *n* = 60) and HHLA2-low (≤5, *n* = 33) groups. *p* values are from the log-rank test. Results for the continuous score and for alternative cutoffs are provided in Table 3 and Table S1.

Over a median follow-up of 111 months (95% CI, 98–120), 12 deaths, 6 disease-specific deaths, and 8 recurrences were observed. In univariable Cox regression, the HHLA2 total score was not associated with OS (hazard ratio 1.17 per 1-point increase, 95% CI 0.85–1.61; *p* = 0.338), DSS (1.86, 0.83–4.18; *p* = 0.132), or RFS (1.19, 0.80–1.77; *p* = 0.394) (Table 3). Dichotomized comparisons showed the same pattern: none of the five candidate cutoffs reached significance for any endpoint, either before or after Bonferroni correction (all adjusted *p* ≥ 0.39; Table S1). At the >5 cutoff used for descriptive comparisons, recurrence occurred in 7 of 60 HHLA2-high (11.7%) and 1 of 33 HHLA2-low patients (3.0%), all-cause death in 9 (15.0%) and 3 (9.1%), and all six disease-specific deaths in the HHLA2-high group (10.0% vs. 0%); Kaplan–Meier curves are shown in Figure 2.

### 3.3. HHLA2 Expression in Breast Cancer Compared to Other Solid Tumors Using TCGA Database

**Figure 3:**
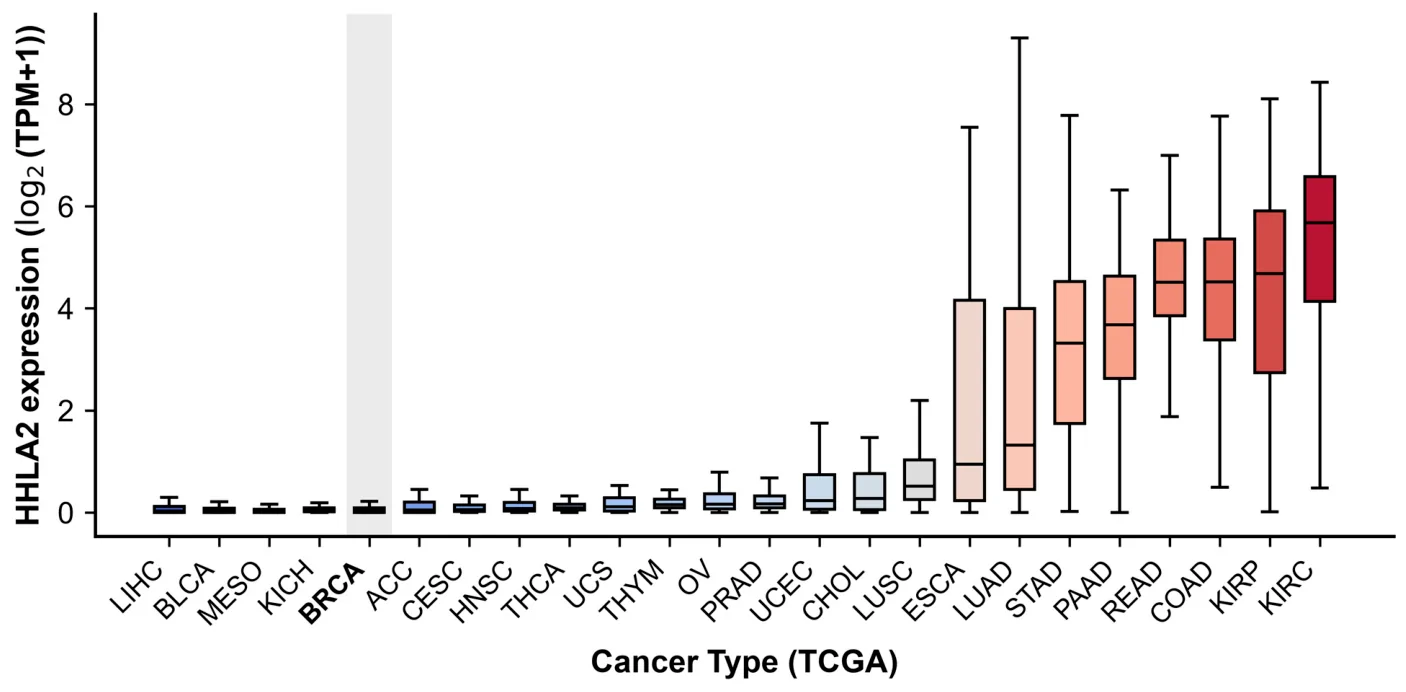
Pan-cancer analysis of HHLA2 expression across 24 solid tumor types in TCGA (8434 primary tumor samples). Tumor types are ordered by increasing median expression. Boxplots show the median and interquartile range of HHLA2 expression (log_2_(TPM+1)). Breast cancer (BRCA, shaded) is among the lowest-expressing types.

To place these findings in a broader context, HHLA2 mRNA expression was compared across 24 solid tumor types in TCGA (8434 primary tumor samples; Figure 3). Breast cancer ranked among the five lowest-expressing types, together with hepatocellular carcinoma (LIHC), urothelial carcinoma of the bladder (BLCA), mesothelioma (MESO) and chromophobe renal cell carcinoma (KICH); medians in this group were closely clustered (0.017–0.028 TPM). The highest expression was observed in clear cell (KIRC) and papillary (KIRP) renal cell carcinoma, followed by colorectal (COAD, READ), pancreatic (PAAD) and gastric (STAD) tumors. Median expression in breast cancer was 0.028 TPM, compared with 50.1 TPM in KIRC—an approximately 1800-fold difference.

### 3.4. HHLA2 Expression According to Sample Type and Molecular Subtype in TCGA-BRCA

**Figure 4:**
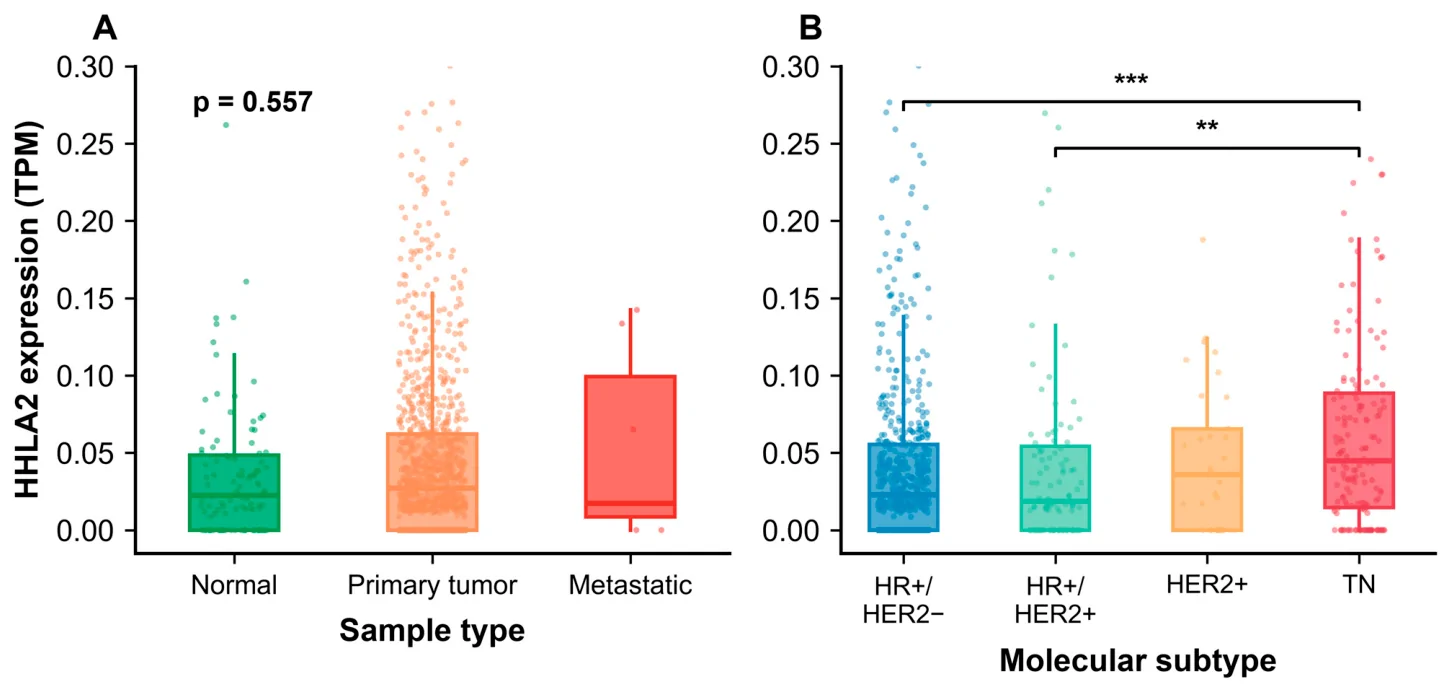
HHLA2 expression in the TCGA-BRCA dataset. (**A**) Expression by sample type (solid tissue normal, *n* = 112; primary tumor, *n* = 1090; metastatic, *n* = 7). (**B**) Expression across molecular subtypes in primary tumors: hormone receptor-positive/HER2-negative (HR+/HER2−, *n* = 613), HR+/HER2+ (*n* = 108), HER2-positive/HR-negative (HER2+, *n* = 33), and triple-negative (TN, *n* = 160). TN tumors showed significantly higher expression than HR+/HER2− and HR+/HER2+ tumors. Overall comparisons were performed using the Kruskal–Wallis test, followed by Dunn’s post hoc test with Benjamini–Hochberg correction for pairwise comparisons (** *p* < 0.01, *** *p* < 0.001). The *y*-axis is truncated at 0.3 for visual clarity, omitting 29 data points in (**A**) and 20 in (**B**) (maximum value: 2.82).

HHLA2 expression was examined in 1209 TCGA-BRCA samples. Expression did not differ significantly across sample types, with comparable levels in normal breast tissue (*n* = 112), primary tumors (*n* = 1090) and metastatic lesions (*n* = 7) (Kruskal–Wallis *p* = 0.557; Figure 4A). In contrast, expression differed significantly by molecular subtype (Kruskal–Wallis *p* = 8.6 × 10^−5^; Figure 4B). Of the 1090 primary tumors, 914 could be assigned a definitive subtype (HR+/HER2−, *n* = 613; HR+/HER2+, *n* = 108; HER2+, *n* = 33; triple-negative [TN], *n* = 160). TN tumors showed higher HHLA2 expression than HR+/HER2− (adjusted *p* = 4.8 × 10^−5^) and HR+/HER2+ tumors (adjusted *p* = 0.0015), whereas the HR+/HER2−, HR+/HER2+ and HER2+ subtypes did not differ significantly from one another. Median expression was 0.045 TPM in TN tumors compared with 0.023, 0.019 and 0.036 TPM in the HR+/HER2−, HR+/HER2+ and HER2+ subtypes, respectively; detectable transcript was present in 80.0% of TN tumors versus 66–68% of the other subtypes. Sequencing depth did not differ across subtypes (Kruskal–Wallis *p* = 0.85). Although detectability increased with depth, the higher detection rate in TN tumors relative to the other subtypes persisted after adjustment (odds ratio 1.88, 95% CI 1.24–2.86; *p* = 0.003).

### 3.5. Prognostic Significance of HHLA2 Expression in TCGA-BRCA Molecular Subtypes

To examine whether the subtype-related differences in HHLA2 expression carry prognostic information, survival analyses were performed separately in the HR-positive and TN cohorts. For Kaplan–Meier display, patients were additionally grouped by detectability (TPM > 0 versus TPM = 0). Median follow-up was 27 months (95% CI, 24–31) in the HR-positive cohort and 33 months (95% CI, 25–39) in the TN cohort.

**Figure 5:**
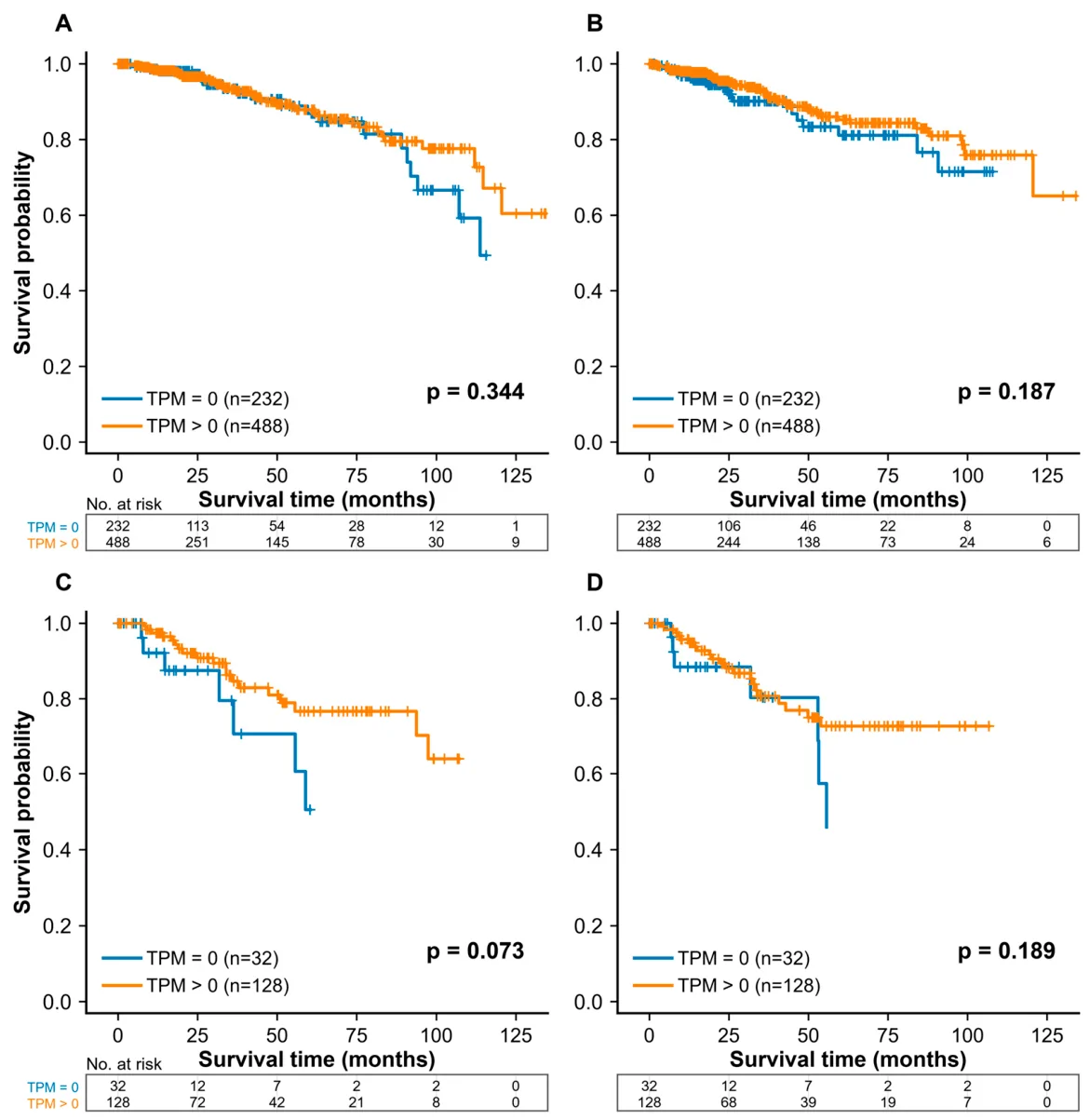
Kaplan–Meier survival analysis in TCGA-BRCA subgroups based on HHLA2 detectability. (**A**) Overall survival (OS) and (**B**) progression-free interval (PFI) in the hormone receptor-positive (HR+) cohort; (**C**) OS and (**D**) PFI in the triple-negative cohort. Events (TPM = 0 vs. TPM > 0) were 22 vs. 43 (OS) and 24 vs. 42 (PFI) in the HR+ cohort, and 8 vs. 19 (OS) and 7 vs. 21 (PFI) in the triple-negative cohort. For display, curves are truncated when fewer than five patients remain at risk; risk tables and log-rank tests were calculated without this truncation. Survival data were obtained from the TCGA Pan-Cancer Clinical Data Resource.

In the HR-positive cohort (*n* = 720), continuous HHLA2 expression was not associated with overall survival (hazard ratio per SD 0.86, 95% CI 0.62–1.20; *p* = 0.375) or the progression-free interval (0.90, 0.67–1.22; *p* = 0.499). In the TN cohort (*n* = 160), point estimates were below unity for both endpoints (0.51, 0.22–1.19; *p* = 0.119 and 0.65, 0.32–1.32; *p* = 0.236) but did not reach significance. Dichotomization by detectability gave concordant results (Figure 5; log-rank *p* = 0.344 and 0.187 in HR-positive, and 0.073 and 0.189 in TN tumors for OS and PFI, respectively), as did median and upper-quartile splits (Table S2).

## 4. Discussion

This study investigated the clinical and prognostic relevance of HHLA2 in HR-positive breast cancer using immunohistochemistry, together with a complementary analysis of the TCGA-BRCA cohort. HHLA2 immunoreactivity was detected in 92.5% of HR-positive breast cancers, yet it showed no significant association with conventional clinicopathologic parameters or survival. In TCGA, HHLA2 transcript levels differed by molecular subtype, being higher in triple-negative than in HR-positive tumors, but this difference was not associated with survival in either subgroup. Notably, the direction of the non-significant survival trends differed between the two cohorts. Across both analyses, HHLA2 expression in breast cancer showed no consistent prognostic association.

In our cohort, HHLA2 expression showed no significant associations with clinicopathologic features including age, tumor size, nodal status, histologic grade, or HER2 positivity. This lack of association is consistent with previous studies in other cancer types where HHLA2 expression was similarly unrelated to clinicopathologic parameters [3,4,5]. However, limited studies have specifically examined HHLA2 in breast cancer. Janakiram et al. reported HHLA2 upregulation in 56% of early-stage TNBC cases (*n* = 50, stage I–III) [9]. In the same study, copy-number gains at the HHLA2 locus were detected in 29% of basal breast cancers, which the authors proposed as a possible basis for increased HHLA2 protein expression [9]. Notably, Janakiram et al. found that high HHLA2 expression in TNBC was significantly associated with nodal involvement and advanced stage [9]. While nodal involvement and advanced stage were numerically more frequent in HHLA2-high tumors in our cohort, these associations did not reach statistical significance. Associations reported in triple-negative disease may not extend to HR-positive tumors, in which HHLA2 is expressed at lower levels.

The prognostic role of HHLA2 remains inconsistent across cancer types. In several malignancies, including colorectal, lung, and pancreatic cancers, high HHLA2 expression has been associated with poor prognosis and shorter overall survival [3,5,15], suggesting a potential immunosuppressive role. However, contrasting findings have emerged from other tumor types, where HHLA2 expression demonstrated either no prognostic impact or was associated with favorable outcomes [4,11]. These conflicting results may reflect differences in tumor-specific immune microenvironments and the dual receptor system of HHLA2. HHLA2 engages the costimulatory receptor TMIGD2 on naïve T and NK cells and the coinhibitory receptor KIR3DL3 on activated T and NK cells [2], and the balance between these interactions may differ across tumor types, potentially contributing to the divergent prognostic associations reported. HR-positive breast cancer is characterized by relatively low immune infiltration, and, unlike in TNBC or HER2-positive disease, higher lymphocytic infiltration in this group has not been consistently associated with better outcome and has even been linked to shorter survival in some series [13,16,17]. A limited prognostic effect of HHLA2 in HR-positive tumors could therefore have been anticipated. However, immune infiltration does not predict whether tumor cells express the ligand, and HHLA2 was in fact detected in the great majority of HR-positive tumors in our cohort. The absence of any survival difference despite immunoreactivity in most tumors indicates that HHLA2 immunoreactivity alone did not identify a prognostic subgroup in this cohort. Given the small number of events, however, smaller but clinically relevant associations cannot be excluded.

Antibodies directed at HHLA2 and at its inhibitory receptor KIR3DL3 have shown activity in preclinical models [2,18] and are under investigation in other solid tumors [19]. Our data do not support extending this approach to HR-positive breast cancer on the basis of ligand immunoreactivity alone. HHLA2 immunoreactivity was detected in most tumors without any association with outcome, and the low immune infiltration of this group would be expected to limit the effect of checkpoint-directed therapy. Membranous expression, the receptors TMIGD2 and KIR3DL3, immune infiltration and treatment response were not assessed in this study, so no inference about therapeutic relevance can be drawn from these findings. Any future evaluation would need to assess these elements together and would more plausibly focus on subtypes with higher expression and richer infiltration, such as TNBC.

Several limitations should be acknowledged. First, the sample size (*n* = 93) and single-center design limited the power to detect associations with outcome, and the small number of events means that the survival analyses should be regarded as exploratory. With 12 deaths, 6 disease-specific deaths and 8 recurrences, the study had 80% power to detect only hazard ratios of ≥5.4, ≥10.9 and ≥7.9, respectively, for the dichotomized comparison (≥2.3–3.1 per SD of the continuous score); smaller but clinically relevant effects cannot be excluded. In addition, treatment records were available for only 24 of the 93 patients; 23 received adjuvant chemotherapy (anthracycline-based in 22) and all 23 with endocrine data received an aromatase inhibitor, but treatment could not be incorporated into the survival analyses. Second, HHLA2 immunoreactivity was detected in 92.5% of tumors in our cohort, with moderate-to-strong intensity in 67.7%, whereas HHLA2 transcript was undetectable in 29.9% of primary tumors in TCGA-BRCA. Because protein and transcript were measured in separate cohorts, this discordance could not be examined directly, and the extent to which HHLA2 immunoreactivity reflects HHLA2 protein in breast tissue warrants confirmation with orthogonal methods. The polyclonal antibody was validated only against an external positive tissue and internal negative structures; reagent negative controls were not included, and membranous expression—the fraction relevant to receptor engagement—was not quantified separately. Nonspecific cytoplasmic staining therefore cannot be excluded and may contribute to the discrepancy between the high IHC positivity and the very low TCGA transcript levels. Third, each case was represented by a single tissue core, which may not capture intratumoral heterogeneity of HHLA2 expression. Fourth, neither tumor-infiltrating lymphocytes nor the HHLA2 receptors TMIGD2 and KIR3DL3 were assessed, so the proposed relationship between immune context and the absence of a prognostic effect could not be tested directly. Finally, the endpoints and follow-up differed between the two cohorts. Recurrence and disease-specific death were ascertained directly in our cohort, over a median follow-up of 111 months. The TCGA analyses instead relied on the progression-free interval, with a median follow-up of 27 months in the HR-positive and 33 months in the triple-negative cohort. Validation in larger, multi-institutional cohorts, together with immune cell profiling by multiplex immunohistochemistry or spatial transcriptomics, would clarify the role of HHLA2 in the breast cancer immune microenvironment.

## 5. Conclusions

HHLA2 immunoreactivity was detected in the majority of HR-positive breast cancers, but no association with clinicopathologic features or outcome was detected. Analysis of TCGA-BRCA showed higher HHLA2 transcript levels in TNBC than in HR-positive tumors, yet this difference did not translate into a prognostic effect in either group. Given the small number of events in our cohort, modest effects cannot be excluded. Its relevance as a therapeutic target remains uncertain and will require studies that assess membranous HHLA2 expression alongside its receptors and the immune infiltrate.:

## Figures and Tables

**Table 1:** Clinicopathologic characteristics according to HHLA2 expression.

Clinicopathologic Parameters	HHLA2 Expression (*n*, %)		*p* Value
	Low (33, 35.5)	High (60, 64.5)	
Age, median (IQR)	56 (49–61)	54 (46–61)	0.329
Tumor size			0.277
≤2 cm	21 (63.6)	30 (50.0)	
>2 cm	12 (36.4)	30 (50.0)	
Node metastasis			0.516
Absent	19 (57.6)	29 (48.3)	
Present	14 (42.4)	31 (51.7)	
Stage			0.267
I	14 (42.4)	20 (33.3)	
II	11 (33.3)	19 (31.7)	
III	8 (24.2)	21 (35.0)	
Tumor grade			0.148
Low	8 (24.2)	24 (40.0)	
Intermediate	18 (54.5)	27 (45.0)	
High	7 (21.2)	9 (15.0)	
HER2 status			0.741
Negative	30 (90.9)	52 (86.7)	
Positive	3 (9.1)	8 (13.3)	

Data are presented as median (interquartile range) for age and as *n* (%) for categorical variables; percentages are calculated within each HHLA2 group. HHLA2-high was defined as a total immunohistochemical score > 5. Age was compared using the Mann–Whitney U test; tumor size, node metastasis and HER2 status using Fisher’s exact test; and stage and tumor grade using the linear-by-linear association test, which takes the ordinal nature of these variables into account.

**Table 2:** Spearman correlation between HHLA2 expression and breast cancer prognostic factors.

Clinicopathologic Parameters	Spearman’s ρ	*p* Value
Tumor size	0.079	0.451
Node metastasis	0.059	0.572
Stage	0.054	0.610
Tumor grade	−0.143	0.172
HER2 status	0.027	0.800

Spearman rank correlation between the HHLA2 total immunohistochemical score (0–8) and each clinicopathologic variable. Tumor size was analyzed as a continuous variable (cm); node metastasis (absent/present) and HER2 status (negative/positive) were coded as binary variables; and stage (I/II/III) and tumor grade (low/intermediate/high) as ordinal variables. Whereas Table 1 compares groups defined by a dichotomized HHLA2 score, this analysis treats the score as a continuous measure.

**Table 3:** Univariable Cox regression of the HHLA2 total immunohistochemical score and survival in the study cohort (*n* = 93).

Endpoint	Events	Hazard Ratio per 1-Point Increase	95% CI	*p* Value
Overall survival	12	1.17	0.85–1.61	0.338
Disease-specific survival	6	1.86	0.83–4.18	0.132
Relapse-free survival	8	1.19	0.80–1.77	0.394

The HHLA2 total immunohistochemical score (intensity plus proportion, range 0–8) was entered as a continuous variable in univariable Cox proportional hazards regression; hazard ratios are expressed per 1-point increase in score. Multivariable analysis was not performed because of the small number of events. CI, confidence interval.

## Data Availability

The TCGA-BRCA RNA-sequencing data analyzed in this study are publicly available from the Genomic Data Commons Data Portal (https://portal.gdc.cancer.gov/), accessed on 18 April 2024. The immunohistochemical and clinical data are not publicly available because they contain information that could compromise patient privacy.

## Supplementary Materials

The following supporting information can be downloaded at https://www.mdpi.com/article/10.3390/biomedicines14092066/s1, Figure S1: External positive control for HHLA2 immunohistochemistry; Table S1: Survival outcomes according to different total immunohistochemical score cutoffs for HHLA2 expression in the study cohort (*n* = 93); Table S2: Survival outcomes in the TCGA-BRCA cohort according to continuous HHLA2 expression and alternative cutoffs.

## Abbreviations

The following abbreviations are used in this manuscript:

DFI	Disease-free interval
DSS	Disease-specific survival
ER	Estrogen receptor
FFPE	Formalin-fixed, paraffin-embedded
HHLA2	Human endogenous retrovirus-H long terminal repeat-associating protein 2
HR	Hormone receptor
IHC	Immunohistochemistry
KIR3DL3	Killer cell immunoglobulin-like receptor, three Ig domains and long cytoplasmic tail 3
OS	Overall survival
PFI	Progression-free interval
PR	Progesterone receptor
RFS	Relapse-free survival
TCGA	The Cancer Genome Atlas
TMA	Tissue microarray
TMIGD2	Transmembrane and immunoglobulin domain containing 2
TNBC	Triple-negative breast cancer
TPM	Transcripts per million.
