# Supplementary material for: Clinical Evaluation of HHLA2 in Hormone Receptor-Positive Breast Cancer: Expression Patterns and Prognostic Implications
Source: Biomedicines. 2026 Sep 15;14(9):2066. doi: 10.3390/biomedicines14092066 (PMC13604577; doi:10.3390/biomedicines14092066)
Supplement: Supplementary file 1 [file biomedicines-14-02066-s001.zip › Supplementary_Figure.pdf]

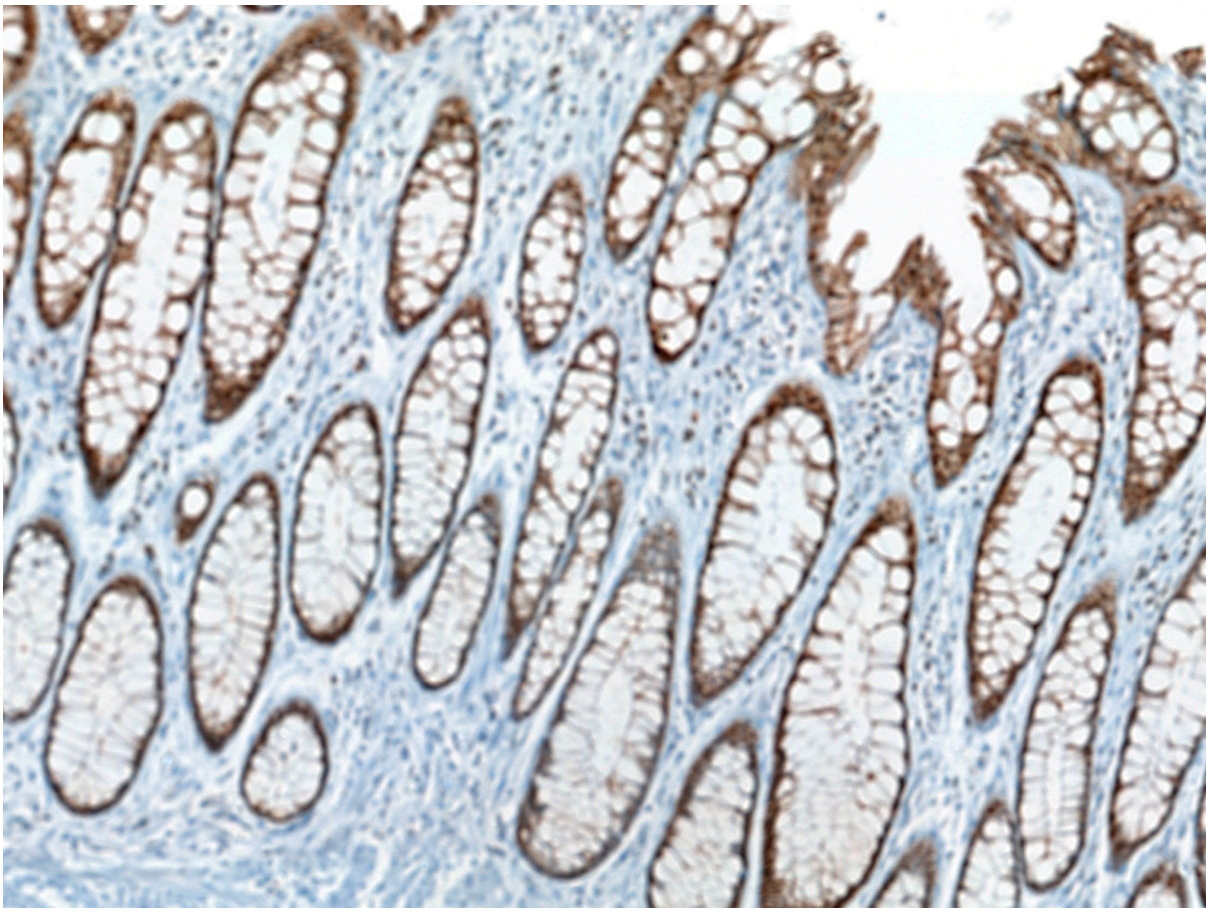

Figure S1. External positive control for HHLA2 immunohistochemistry. Normal colonic mucosa shows strong cytoplasmic HHLA2 immunoreactivity in crypt epithelium, with negative lamina propria stroma. Same antibody, dilution and platform as in the study cohort. Original magnification, ×200.
